# The Swedish Stroke Self-Efficacy Questionnaire: translation and cross-cultural adaptation

**DOI:** 10.1186/s41687-024-00735-7

**Published:** 2024-06-05

**Authors:** Erika Klockar, Maya Kylén, Linnea McCarthy, Lena von Koch, Catharina Gustavsson, Fiona Jones, Marie Elf

**Affiliations:** 1 School of Health and Welfare, Dalarna University, Högskolegatan 2, Falun, Sweden; 2 Department of Health Sciences, Lund University, Lund, Sweden; 3 Department of Neurobiology, Care Sciences and Society, Karolinska Institutet, Stockholm, Sweden; 4 Theme Neuro, Karolinska University Hospital, Stockholm, Sweden; 5 Center for Clinical Research Dalarna, Falun, Sweden; 6 Department of Public Health and Caring Sciences, Uppsala University, Uppsala, Sweden; 7 Faculty of Health and Social Care Sciences, Kingston University & St George’s, University of London, London, UK

**Keywords:** Self-efficacy, Stroke, Questionnaire, Cross-cultural, Rehabilitation

## Abstract

**Objective:**

To translate and cross-culturally adapt the Stroke Self-Efficacy Questionnaire (SSEQ) from English to Swedish and to evaluate psychometric properties of the questionnaire.

**Methods:**

A cross-sectional study design, where the translation followed a process including initial translation, synthesis, backward translation, expert committee, and pretest. Content validity was assessed using Content validity index (CVI). Psychometric assessments included floor-ceiling effects and internal consistency.

**Results:**

Language and cultural congruence were achieved, and content validity index scores were high (0.923-1). The psychometric evaluations provided acceptable outcomes concerning internal consistency, with Cronbach’s alpha scores for the total scale (0.902), the activities subscale (0.861) and the self-management subscale (0.818) respectively. Ceiling effects were evident, but no floor effects.

**Conclusion:**

This study found the Swedish version of the SSEQ promising as a tool for assessment of self-efficacy in a Swedish stroke care setting, although further psychometric assessments are recommended in future studies.

**Supplementary Information:**

The online version contains supplementary material available at 10.1186/s41687-024-00735-7.

## Background

The profound impact of stroke on an individual’s life has been extensively documented, revealing various degrees of long-term physical, cognitive and communication impairments and persisting functional, emotional and social challenges [1]. Some of these issues, such as memory and planning difficulties, fatigue, and post-stroke depression, can be invisible but significantly affect a person’s well-being [2, 3]. Thus, it is critical to explore how people navigate life after stroke and their confidence to reintegrate into everyday living. The long-term consequences of stroke also necessitate that the rehabilitation process continues well after discharge from the hospital, and individuals require supportive ways to learn skills to manage longer term [4]. Studies have highlighted that people who experience a stroke may face inadequate preparation, insufficient information, and limited support during and after discharge [5]. Consequently, they often feel unprepared and have low confidence in resuming their previous valued activities [6].

Self-efficacy is a fundamental construct that sheds light on how individuals develop confidence to navigate challenges and setbacks and increase feelings of capability in their everyday lives. It is a cornerstone in Albert Bandura’s Social Cognitive Theory [7], defined as ‘the belief in one’s capabilities to organize and execute the necessary actions to achieve desired outcomes.’ Self-efficacy is a context-specific construct influenced by various factors, including vicarious experiences, mastery experiences, psychological states and verbal persuasion [8]. Vicarious experiences involve observing relatable individuals who succeed in a particular behavior, which can significantly enhance self-efficacy. Mastery experiences—to successfully carry out tasks through our own efforts—is one of the strongest sources of self-efficacy. Verbal persuasion has a weaker effect but can raise self-efficacy when individuals receive support and encouragement from credible and trusted sources, while psychological states, such as anxiety or feelings of helplessness, can lower self-efficacy. Bandura’s work also emphasizes the interplay among behavior, environment, and personal factors (especially self-efficacy) in driving behavioral change [7]. This dynamic relationship is often depicted as a triangle, with each cornerstone influencing the others [8].

Studies have shown that levels of self-efficacy can predict quality of life and functional independence for persons post-stroke [9]. High levels of self-efficacy have been associated with setting more advanced goals, reaching greater performance success, and being more resilient in challenging situations [10]. Lower levels of self-efficacy, on the other hand, have been linked to reduced motivation and higher levels of depression and anxiety [11] and reduced change in balance and motor function following rehabilitation [12]. Overall, self-efficacy correlates strongly with mobility, activity, and higher performance levels in individuals with stroke [10]. Since self-efficacy also influences the initiation of behavior change [7], it is considered an essential contributor to supporting self-management after stroke [13]. Self-management interventions often aim to enable individuals to gain the knowledge and skills to actively manage their rehabilitation or recovery by increasing their confidence and motivation [4]. Today, self-management support is considered an important factor in person-centered care and in the rehabilitation of long-term conditions [14].

Accurately assessing self-efficacy after a stroke is crucial for gaining valuable insights into the factors contributing to successful rehabilitation or hindering progress in achieving functional and goal-oriented outcomes [11, 13, 15]. Such information may direct rehabilitation efforts toward areas of utmost importance to individuals and identify activities where self-efficacy may be lower. It may also aid in identifying factors that can influence and change self-efficacy, thereby contributing to the design of stroke rehabilitation with a self-management focus [13].

A general self-efficacy scale exists but given the situation-specific nature of self-efficacy, a designated measurement of self-efficacy after stroke has been viewed as necessary [16]. The Stroke Self-Efficacy Questionnaire (SSEQ), developed in the United Kingdom in 2008 (Supplementary 1), and updated in 2014, is a self-reported measure designed to assess self-efficacy in relevant areas of functioning post-stroke [13, 16]. The SSEQ consists of 13 items with two primary constructs: (1) Activities, which are based on common functional difficulties experienced after a stroke (8 items), and (2) Self-management (5 items). Respondents rate their confidence using a four-point Likert scale ranging from 0 = not at all confident to 3 = very confident. The total score ranges from 0 to 39, with a higher score indicating a stronger perceived self-efficacy. To calculate the final score, the sum of the filled items is divided by the number of items completed. The scale has demonstrated good internal consistency, criterion validity [16], and construct validity [13]. The questionnaire has undergone Rasch analysis to further evaluate its psychometric properties, and confirmed that the two subscales showed good construct validity and the items yielded satisfying results according to the requirements for Rasch analysis [13]. The SSEQ has been translated into several languages, including Danish, Portuguese, Turkish, Hausa, Italian, Brazilian, Chinese, and Arabic [3, 6, 9, 17–23]. These translations have overall been successful in their validation and cross-cultural adaptations.

Currently, no specific scale is available in Swedish to assess self-efficacy among persons post-stroke. A Swedish version of the SSEQ could provide a valuable tool for assessing self-efficacy in this population. Therefore, this study aimed to translate and cross-culturally adapt the SSEQ from English to Swedish (SSEQ-SWE) and assess the questionnaire’s psychometric properties.

## Methods

### Study design

**Fig. 1:**
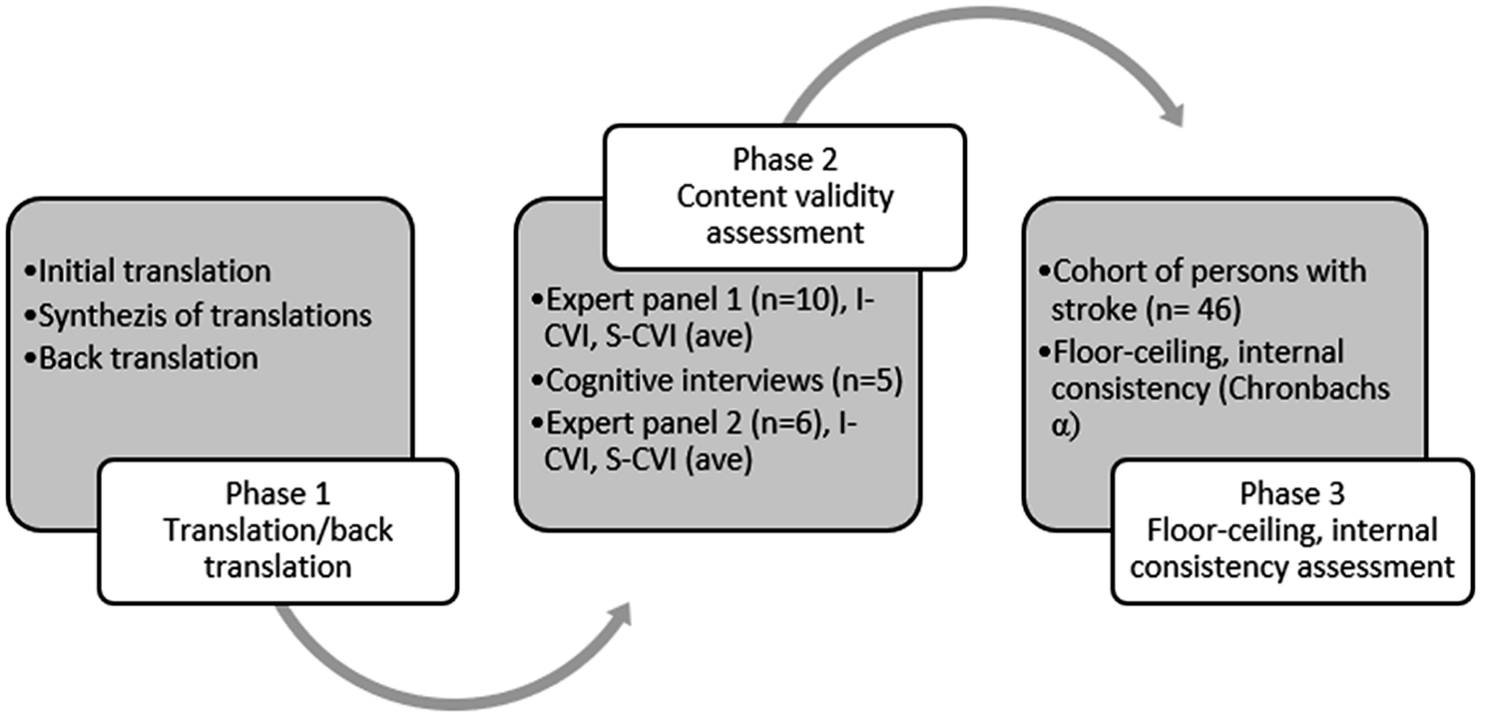
The process of translation and cross-cultural adaption of SSEQ to Swedish

The original instrument developer (co-author FJ) gave permission to translate and cross-culturally adapt the instrument to a Swedish context. The process followed a three-phase cross-sectional study design (Fig. 1).

### Participants

To recruit expert panels in phase 2 we reached out to a reference group in an ongoing stroke research project [24]. Purposeful sampling was applied to obtain a range of views from different areas—from persons with lived experience of stroke, from health care professionals (HCP: s) in stroke care and from researchers within the field of stroke or instrument development and translation. The first expert panel included three persons with stroke, two HCP:s, and five researchers. In addition, five persons with stroke were recruited via a stroke association to participate in cognitive interviews. The second expert panel included six community-living individuals with stroke who were recruited through a local stroke association.

In phase 3, we included a cohort of persons with stroke (*n* = 46) that were already recruited from two hospitals in southern Sweden into a larger self-management intervention study [24]. This convenience sample gave us an opportunity to test the instrument in a real-world setting. The inclusion criteria to the intervention study were adults with a stroke diagnosis, discharged from the hospital after receiving stroke care, and capable of comprehending the study goals and procedures. Data were collected on participant’s demographics, function as per the Stroke Impact Scale [25] and self-efficacy as per the Self-efficacy scale [26]. All participants were given written information about the study and provided full informed consent to participate. The study was approved by The Swedish Ethical Review Authority (dnr 2020–02116).

### Procedure for data collection and analyses

We followed the procedure described by Beaton et al. [27], including initial translation, synthesis of translations, back translation, expert panel review, and pretesting of the final version. An iterative approach involved multiple rounds of data collection, analysis, and interpretation, with each round building upon the previous one.

### Phase 1—Translation and back-translation

#### Initial translation

The research group discussed the core constructs of the instrument. Two authors (EK, LM), with clinical stroke experience and Swedish as their native language, independently translated the English version of the SSEQ into Swedish. The focus was on capturing the conceptual meaning rather than a literal translation.

#### Synthesis of translations

The research group compared and discussed the translations to reach a consensus on the most appropriate translation that preserved the conceptual meaning and language. A first version of SSEQ-SWE was agreed upon.

#### Back translation

The first version was back-translated into English by a bilingual professional translator with English as her native language, blinded to the original instrument. A comparison was made between the back-translated items and the original items. Conceptually problematic items were identified and discussed by the research group and the translator, resulting in a new modified version.

### Phase 2 content validity assessment

To assess the relevance of the content of the items, we used two methods—Content Validity Index (CVI) [28] and cognitive interviews. Item content validity (I-CVI) and average scale content validity (S-CVI (ave) were calculated based on expert group ratings. I-CVI was determined for each item by dividing the number of individuals rating the item as quite relevant or highly relevant by the total number of individuals rating the item. S-CVI (ave) was calculated by summing and dividing the average I-CVI values by the number of items. The cutoff score for CVI on the scale level was set at 0.8 and 0.78 on the item level, following Polit & Beck [29].

The two expert panels rated S-CVI (ave) and I-CVI. Additionally, the panel members provided feedback on the items’ phrasing and concerns about the instrument’s form, layout, and legibility. The material from these ratings was considered and discussed within the research group and used to revise wording when deemed appropriate.

Five participants with stroke took part in cognitive interviews guided by open-ended questions about SSEQ-SWE. The interviews focused on assessing how the participants understood the instrument and to get a deeper understanding of its relevance, clarity, and overall suitability. The participants had access to the questionnaire before and during the interview, which was conducted via telephone. ME conducted the interviews and took notes for documentation. Data were analysed by ME and EK with content analysis at a manifest level [30].

### Phase 3 floor-ceiling and internal consistency assessment

The final version of SSEQ-SWE (Supplementary 2) was administered to 46 persons with stroke, discharged from two stroke units in southern Sweden. Descriptive statistics were calculated. Floor- and ceiling effects were measured, and Cronbach α was used to assess internal consistency by calculating the pairwise correlations between items in the SSEQ-SWE. The internal consistency was determined acceptable at values of 0.70 to 0.95 [31]. The statistics were calculated using IBM SPSS Statistics for Windows, Version 28.0 [32].

## Results

### Phase 1 translation and back-translation

The translation of SSEQ to SSEQ-SWE demonstrated overall high consistency between the original version and the backward translation. Nevertheless, certain inconsistencies emerged that required further exploration and discussion, to address the risk that the Swedish translation had inadvertently changed the conceptual meaning of the original items. For instance, discrepancies arose in the interpretation of ‘discharged from therapy,’ which needed to be interpreted differently based on Sweden’s healthcare structure. An agreement was reached at ‘without support from health care’, which was in line with the original intention of the item. Another cultural discrepancy was noted concerning the reference to wall-to-wall carpets, which are uncommon in Sweden. Examples demonstrating varying interpretations of original items by four researchers are provided in Supplementary 3.

### Phase 2 content validity assessment

Data were generated from the first expert panels CVI ratings and free text comments. The S-CVI (ave) was computed at 0.923. I-CVI scores exceeded the threshold of 0.78, except for item 6 (0.75), item 11 (0.625) and item 13 (0.625). These items underwent additional consideration to improve these items.

Data from the cognitive interviews was categorized into scale-level and item-level insights. Overall participants regarded the scale as meaningful and coherent. Moreover, some suggested that administering the scale multiple times could enable tracking confidence of their post-stroke progress. Another reason for administrating the scale on several occasions was that the participants expressed how different phases of recovery involve different needs and goals, and to follow these changes it can be important to measure them repeatedly.

At the item level, results were classified according to the SSEQ’s subcategories: ‘activities’ (items 1–8) and ‘self-management’ (items 9–13). In the subcategory ‘activities’, most items were recognized as genuinely important, with stroke survivors relating these items to personal experiences, indicating their relevance for persons with stroke. Some participants expressed that even though the subscale activities concern practical issues in daily life, it was not just about the activity in itself, but also to have the courage to do it. For example, walking outdoors was not just a matter of being able to manage pavements or rough terrain, but also to have the confidence to leave the house, which could be associated with anxiety. To be surrounded by people could be frightening and a fear of recurrent stroke being outside was also expressed. This indicates that it might not just be the activity that a person reflects upon when answering the questionnaire, but also the feelings, concerns and context that are associated with the activity.

The subcategory ‘self-management’ generated more extensive comments and discussions compared to the subcategory ‘activities’. The interviews stimulated reflections on the support they received and encouraged them to articulate their needs. Several participants expressed when discussing item 10—conduct exercise—that they lacked motivation and did not know how to find it again. They expressed a need to discuss goal setting and motivation with health care professionals to ensure that their rehabilitation exercises would be continued. An example was to be asked “what do you need”—and that this need was not so often practical things connected to exercise but more questions of motivation and goal setting. For some persons, the continuance of rehabilitation meant that they had to coordinate their own care into next care level (from specialist care to community care) which was experienced as exhausting at times and could affect motivation for rehabilitation exercises.

An important issue raised in the cognitive interviews was that the term “effective” from item 13 was interpreted as somewhat negative and raised feelings of a demand of doing things rapidly that took away the joy they felt about managing overall. The term effective was replaced with “faster” in the final version of the instrument. Overall, there were positive attitudes towards being asked these kinds of questions, and as one participant expressed: “*if only I had received these kinds of questions when I was ill!*”.

After some minor rephrasing of the SSEQ-SWE based on the first expert panel and the cognitive interviews, our second expert panel performed CVI rating at scale and item level. Both the S-CVI (ave) and I-CVI attained a score of 1, which indicated strong content validity at scale and item levels. No further adjustments were made.

### Phase 3 floor-ceiling and internal consistency assessment

A total of 46 participants were recruited for pre-test of the final version and at the same time floor-ceiling and internal consistency assessments were performed. Participant characteristics are outlined in Table 1. Out of these, 43 participants completed the questionnaires, while 3 participants left them blank and were excluded from the result.

**Table 1:** Participant characteristics

Characteristics	Participants (*n* = 43)
Sex, %	
Male	54
Female	46
Age, mean, (SD), range	70, (13), 35–91
Days since stroke, mean	67
Education, %	
Primary school	24
High school	38
Higher education, < 3 y	13.3
Higher education, > 3 y	24
Occupation, %	
Retired	69
Employed	16
Sick leave, > 3 months	13
Other arrangement	2
Living arrangements, %	
Living alone	42
Living together	58
Home service, %	
Yes	20
No	80
Location, %	
City	9
Urban area	76
Country side	15
Stroke impact scale	
Activities domain 10–100, mean (SD)	77.92 (22.75)
Recovery 0–100, mean (SD)	66.8 (24.9)
Self-efficacy scale	
Total score (18–180) mean, (SD)	144.9 (33.0)

In Table 2, mean scores, Cronbach’s α and ceiling effects are presented. Ceiling effects were apparent in both subscales. 62% of the answers on the subscale ‘activities’ and 30% of the responses on the subscale ‘self-management’ scored 3 points (very certain) respectively. No floor effects were detected on either of the subscales.

Psychometric analyses of the SSEQ-SWE revealed the following internal consistency scores: total SSEQ-SWE (0.902), activities subscales (0.861), and self-management subscale (0.818). On the subscale ‘activities’, item 6 “Use both your hands for eating your food”, had item-total correlation of 0.511. Calculations showed that if item 6 was to be removed, Cronbach’s α would increase from 0.861 to 0.867. Following discussions within the research group, it was decided to retain item 6 in the questionnaire, based on the assessment that removing the item would result in only a minor change in Cronbach’s alpha.

**Table 2:** Mean score, Cronbach´s α, ceiling effect

Scale	Completed (*n*)	Items (*n*)	Mean score	Cronbach’s α	Ceiling effect
SSEQ-SWE total	43	13	2.30	0.902	50%
SSEQ-SWE act.	43	8	2.45	0.861	62%
SSEQ-SWE sm.	43	5	2.06	0.818	30%

## Discussion

This paper describes the process of translating and cross-culturally adapting the SSEQ questionnaire from its original English version into Swedish language and context, the SSEQ-SWE. The translation process followed Beaton’s framework [27]; a standardized model that is developed for cross-cultural adaptation of patient reported outcome measures and employed in other translations of this questionnaire, for example Kristensen & Pallesen [22]. By adhering to a standardized translation procedure, we aimed to ensure comprehensiveness in all essential steps. Furthermore, we conducted similar psychometric tests as other translation studies—where floor-ceiling calculations and internal consistency has been reported in several other translations of the SSEQ, for example in Danish [22], Turkish [3] and Portuguese [6], ensuring comparability.

Although the back-forward translation was relatively straightforward, challenges still emerged during the adaptation process. Certain terms showed subtle nuances and divergent meanings between English and Swedish. Addressing these discrepancies demanded careful consideration and attention to ensure accurate translation while preserving the purpose of SSEQ. For instance, item 10 refers to one’s capability to independently perform exercises, rather than solely adhering to a structured rehabilitation program. During the back translation process, it became evident that the term “exercise” carries varying semantic interpretations in translation. Cultural variations were also notably obvious when translating items associated with aspects of how care is organized, especially those connected to extended care responsibilities (item 9). The cross-cultural adaptation of item 9 has been discussed in other translations as well [3, 19, 22, 23], underscoring the necessity for a comprehensive adaptation of the questionnaire to the specific usage context.

The active involvement of the expert panels, including persons with stroke, researchers and healthcare professionals enabled comprehensive exploration of culturally sensitive items. The panel’s diverse expertise contributed to the comprehensive translation, which was reflected in the high CVI scores on both item and scale level. The individuals who participated in the interviews contributed with different views and feedback on the questionnaires as a whole and its subscales. The large number of remarks on the subscale ‘self-management’ indicated that these items were particularly significant for persons with stroke. Many comments from people with stroke concerned their own experiences of care and identified gaps in self-management support, implying the importance of such questions.

In accordance with other translations [3, 6, 22], we found ceiling effects but no floor effects in the questionnaire. These findings were to some extent anticipated in the ‘activities’ subscale due to the homogeneity in post-stroke functioning in the cohort, where most participants rated their activity level relatively high as per the Stroke Impact Scale. The ‘self-management’ subscale demonstrated a smaller ceiling effect, indicating that this construct may exhibit greater variation regardless of functioning levels. This result is somewhat contrasting to the result from the Italian translation, where the participants showed lower levels of confidence in the activity subscale but scored higher in the self-management subscale [19]. This may be due to additional cultural differences which illustrates the importance of a thorough translation process to ensure that an instrument is adapted to the context in which it is being used.

Overall, the psychometric properties analysis of SSEQ-SWE demonstrated good internal consistency, which is in line with previous translations of the questionnaire [13, 22, 23]. This suggests that the items are interrelated and measure the intended construct. However, while Cronbach’s alpha is a widely used measure for internal consistency, future studies should employ more comprehensive psychometric analyses to ensure its robustness in a Swedish healthcare context.

Furthermore, studies employing focus groups or think aloud interviews could extend the qualitative understanding of the instrument, that were not explored in full using a cognitive interview approach, as in this study.

The participants in our study were homogeneous in terms of health status, which is a limitation, whereas other participant characteristics showed larger variation, such as gender and age. The wide spectrum of post stroke functional deficits may not have been adequately covered within our cohort. Hence, future studies should encompass a more diverse cohort to cover a broader range of post stroke functioning to ensure the applicability of the questionnaire across different populations of people with stroke.

Despite being developed over 15 years ago, and considering the advancements in acute stroke care, the items seem to remain relevant. This is supported by the participants who during the cognitive interviews highlighted the importance of healthcare professionals posing the specific questions present in SSEQ.

In conclusion, the translating and cross-cultural adaption process of the SSEQ to SSEQ-SWE appears to be successful regarding face validity and internal consistency. It has the potential to be used as a practical tool in stroke care in Sweden, although further psychometric assessments are required. Understanding self-efficacy is valuable in evaluating confidence to manage after discharge from rehabilitation. It may also support evaluation of self-efficacy and the quality and effectiveness of stroke self-management interventions.

### Electronic supplementary material

Below is the link to the electronic supplementary material.

Supplementary Material 1

Supplementary Material 2

Supplementary Material 3

## Data Availability

Data are available from the corresponding author on a reasonable request.

## Abbreviations

CVI: Content Validity Index
I-CVI: Item Content Validity Index
S-CVI (ave): Scale Content Validity Index (average)
SD: Standard Deviation
SSEQ: Stroke Self-efficacy Questionnaire
SSEQ-SWE: Stroke Self-efficacy Questionnaire– Swedish version
