# Supplementary material for: The Swedish Stroke Self-Efficacy Questionnaire: translation and cross-cultural adaptation
Source: J Patient Rep Outcomes. 2024 Jun 5;8:55. doi: 10.1186/s41687-024-00735-7 (PMC11153470; doi:10.1186/s41687-024-00735-7)
Supplement: Supplementary file 1 — Supplementary Material 1 [file 41687_2024_735_MOESM1_ESM.docx]

*Supplementary 1, Stroke Self-efficacy questionnaire*

The STROKE SELF-EFFICACY QUESTIONNAIRE

These questions are about your confidence that you can do some tasks that may have been difficult for you since your stroke.

For each of the following tasks, please circle a point on the scale that shows how confident you are that you can do the tasks now in spite of your stroke.

Where 0 = *not at all confident* and 3 = *very confident*

Not at all Very

confident confident

0 3

How *Confident* are you *now* that you can

1. Get yourself comfortable in bed every night.

Not at all Very

confident confident

0 3

2. Get yourself out of bed on your own even when you feel tired.

Not at all Very

confident confident

0 3

3. Walk a few steps on your own on any surface inside your house.

Not at all Very

confident confident

0 3

4. Walk about your house to do most things you want.

Not at all Very

confident confident

0 3

5. Walk safely outside on your own on any surface.

Not at all Very

confident confident

0 3

6. Use both your hands for eating your food.

Not at all Very

confident confident

0 3

7. Dress and undress yourself even when you feel tired.

Not at all Very

confident confident

0 3

8. Prepare a meal you would like for yourself.

Not at all Very

confident confident

0 3

9. Persevere to make progress from your stroke after

discharge from therapy.

Not at all Very

confident confident

0 3

10. Do your own exercise programme every day.

Not at all Very

confident confident

0 3

11. Cope with the frustration of not being able to do

some things because of your stroke.

Not at all Very

confident confident

0 3

12. Continue to do most of the things you liked to do

before your stroke.

Not at all Very

confident confident

0 3

13. Keep getting faster at the tasks that have been

slow since your stroke.

Not at all Very

confident confident

0 3
