# Supplementary material for: The Swedish Stroke Self-Efficacy Questionnaire: translation and cross-cultural adaptation
Source: J Patient Rep Outcomes. 2024 Jun 5;8:55. doi: 10.1186/s41687-024-00735-7 (PMC11153470; doi:10.1186/s41687-024-00735-7)
Supplement: Supplementary file 2 — Supplementary Material 2 [file 41687_2024_735_MOESM2_ESM.docx]

*Supplementary 2, SSEQ-SWE*

**SSEQ – SWE Stoke Self-Efficacy Questionnaire Swedish version**

**Frågeformulär om tilltro till den egna förmågan i vardagslivet efter stroke**

Nedan följer ett antal påståenden som handlar om hur säker du känner dig på att göra vissa saker som kan vara svåra efter din stroke. Påståendena handlar om vad du tror att du skulle kunna göra om du försökte, även om du inte har försökt ännu. Det är vad du tror om din förmåga just nu att kunna utföra uppgifterna du skall tänka på.

Sätt ett kryss i rutan med den siffra som motsvarar hur säker du känner dig just nu på att utföra de olika uppgifterna trots din stroke.

0 betyder mycket osäker och 3 betyder mycket säker

Mycket osäker Ganska osäker Ganska säker Mycket säker

| 0 |  | 1 |  | 2 |  | 3 |
| --- | --- | --- | --- | --- | --- | --- |

**Hur säker känner du dig just nu på att:**

**1. Kunna lägga dig till rätta i sängen på egen hand**

Mycket osäker Ganska osäker Ganska säker Mycket säker

| 0 |  | 1 |  | 2 |  | 3 |
| --- | --- | --- | --- | --- | --- | --- |

*Förklaring: Hur säker känner du dig på att du skulle kunna hitta en bekväm ställning i sängen utan hjälp av någon annan, trots de rörelsesvårigheter som du kanske har.*

**2. Ta dig ur sängen på egen hand**

Mycket osäker Ganska osäker Ganska säker Mycket säker

| 0 |  | 1 |  | 2 |  | 3 |
| --- | --- | --- | --- | --- | --- | --- |

*Förklaring: Hur säker känner du dig på att du skulle kunna ta dig ur sängen på egen hand, även om du känner dig trött eller har en dålig dag?*

**3. Gå några steg på olika underlag i din bostad**

Mycket osäker Ganska osäker Ganska säker Mycket säker

| 0 |  | 1 |  | 2 |  | 3 |
| --- | --- | --- | --- | --- | --- | --- |

*Förklaring: Hur säker känner du dig just nu på att du kan gå några steg utan hjälp av någon annan person, på olika typer av golv, över trösklar och på mattor i ditt hem? Det kan vara med eller utan gånghjälpmedel.*

**4. Ta dig runt i din bostad och göra det som du tycker om att göra på egen hand**

Mycket osäker Ganska osäker Ganska säker Mycket säker

| 0 |  | 1 |  | 2 |  | 3 |
| --- | --- | --- | --- | --- | --- | --- |

*Förklaring: Hur säker känner du dig på att du skulle kunna ta dig till de rum du vill för att göra sådant du vill, till exempel komma åt särskilda saker, ta fram föremål ur skåp och liknande?*

**5. Gå utomhus på olika underlag på egen hand**

Mycket osäker Ganska osäker Ganska säker Mycket säker

| 0 |  | 1 |  | 2 |  | 3 |
| --- | --- | --- | --- | --- | --- | --- |

*Förklaring: Även om du inte prövat ännu, hur säker känner du dig att du skulle kunna gå utan hjälp av någon annan person på till exempel gräs, grus eller trottoarer? Det kan vara med eller utan gånghjälpmedel.*

**6. Använda båda dina händer vid måltider**

Mycket osäker Ganska osäker Ganska säker Mycket säker

| 0 |  | 1 |  | 2 |  | 3 |
| --- | --- | --- | --- | --- | --- | --- |

*Förklaring: Om en arm är svagare efter din stroke, hur säker är du på att du skulle kunna involvera handen vid måltider för att till exempel hålla i en tallrik, greppa ett glas eller kopp, använda bestick eller liknande?*

**7. Klä på och av dig på egen hand**

Mycket osäker Ganska osäker Ganska säker Mycket säker

| 0 |  | 1 |  | 2 |  | 3 |
| --- | --- | --- | --- | --- | --- | --- |

*Förklaring: Hur säker är du på att du skulle kunna klä på och av dig på egen hand, även när du känner dig trött eller har en dålig dag?*

**8. Göra i ordning en måltid som du vill äta**

Mycket osäker Ganska osäker Ganska säker Mycket säker

| 0 |  | 1 |  | 2 |  | 3 |
| --- | --- | --- | --- | --- | --- | --- |

*Förklaring: Om du tänker på en måltid du tycker om, hur säker känner du dig på att du skulle kunna värma eller tillaga den på egen hand? Det kan vara med eller utan hjälpmedel.*

**9. Fortsätta försöka göra framsteg efter din stroke även utan stöd från vården**

Mycket osäker Ganska osäker Ganska säker Mycket säker

| 0 |  | 1 |  | 2 |  | 3 |
| --- | --- | --- | --- | --- | --- | --- |

*Förklaring: Hur säker känner du dig på att fortsätta göra framsteg från din stroke, även när du skrivs ut från sjukhus eller rehabiliteringsenhet? Till exempel genom att på egen hand sätta mål för att göra aktiviteter som du ännu inte klarat och utmana dig själv att klara nya saker.*

**10. Kunna genomföra din egen träning som planerat**

Mycket osäker Ganska osäker Ganska säker Mycket säker

| 0 |  | 1 |  | 2 |  | 3 |
| --- | --- | --- | --- | --- | --- | --- |

*Förklaring: Hur säker känner du dig på att du klarar att genomföra din träning eller dina rehabiliteringsövningar på egen hand, så ofta som du vill?*

**11. Kunna hantera frustration som kan uppstå om du inte kan göra saker som du kunde göra tidigare**

Mycket osäker Ganska osäker Ganska säker Mycket säker

| 0 |  | 1 |  | 2 |  | 3 |
| --- | --- | --- | --- | --- | --- | --- |

*Förklaring: Om du hamnar i en situation där du inte klarar av vissa saker till följd av din stroke, till exempel att gå, använda din svaga hand, koncentrera dig eller använda mobiltelefon, hur säker känner du dig på att du skulle kunna hantera den frustration som kan uppstå?*

**12. Fortsätta att göra de saker du tyckte om att göra innan du fick stroke**

Mycket osäker Ganska osäker Ganska säker Mycket säker

| 0 |  | 1 |  | 2 |  | 3 |
| --- | --- | --- | --- | --- | --- | --- |

*Förklaring: Tänk på en hobby eller aktivitet du verkligen tyckte om att göra innan du fick stroke, som till exempel att cykla, pyssla i trädgården, snickra, laga mat eller dansa, och fundera kring hur säker du känner dig på att du skulle kunna klara av att göra denna aktivitet.*

**13. Fortsätta bli snabbare på att göra saker som kanske gått långsammare sedan du fick din stroke**

Mycket osäker Ganska osäker Ganska säker Mycket säker

| 0 |  | 1 |  | 2 |  | 3 |
| --- | --- | --- | --- | --- | --- | --- |

*Förklaring: Hur säker känner du dig på att fortsätta försöka bli snabbare och mer effektiv på att till exempel gå, klä på dig, läsa, sköta din* *hygien?*
