# Supplementary material for: The Swedish Stroke Self-Efficacy Questionnaire: translation and cross-cultural adaptation
Source: J Patient Rep Outcomes. 2024 Jun 5;8:55. doi: 10.1186/s41687-024-00735-7 (PMC11153470; doi:10.1186/s41687-024-00735-7)
Supplement: Supplementary file 3 — Supplementary Material 3 [file 41687_2024_735_MOESM3_ESM.docx]

*Supplementary 3. Examples of items and their different interpretations from four different researchers in the group.*

| Persevere to make progress from your stroke after discharge from therapy. | Fortsätta att göra framsteg i din rehabilitering även efter utskrivning  Fortsätta att förbättras från din stroke efter du skrivits ut från sjukhuset  Fortsätta att försöka förbättras efter din stroke även efter hemkomst från sjukhuset  Försöka förbättras efter din stroke efter hemkomst från sjukhuset |
| --- | --- |
| Walk a few steps on your own on any surface inside your house. | Gå några steg på egen hand på olika underlag i huset  Gå några steg självständigt på alla underlag inne i din bostad  På egen hand kan gå några steg på olika underlag (*ytor, utrymmen*) i din bostad  Gå några steg på egen hand (*på olika underlag,* *ytor, utrymmen*) i din bostad |
